# Relationship Between Sleep Disturbances and In Vitro Fertilization Outcomes in Infertile Women: A Systematic Review and Meta‐Analysis

**DOI:** 10.1002/brb3.70293

**Published:** 2025-02-09

**Authors:** Farangis Habibi, Roya Nikbakht, Shayesteh Jahanfar, Mohammad Ahmadi, Maryam Eslami, Marzieh Azizi, Zohreh Shahhosseini

**Affiliations:** ^1^ Midwifery Counseling, Student Research Committee, Nasibeh School of Nursing and Midwifery Mazandaran University of Medical Sciences Sari Iran; ^2^ Department of Biostatistics and Epidemiology, School of Health Mazandaran University of Medical Sciences Sari Iran; ^3^ Department of Public Health and Community Medicine Tufts University School of Medicine Boston Massachusetts USA; ^4^ Behshahr Healthcare Network Mazandaran University of Medical Sciences Sari Iran; ^5^ Imam Khomeini Hospital Complex Tehran University of Medical Sciences Tehran Iran; ^6^ Sexual and Reproductive Health Research Center Mazandaran University of Medical Sciences Sari Iran

**Keywords:** infertility, in vitro fertilization, sleep disorder

## Abstract

**Background:**

In vitro fertilization (IVF) has been acknowledged as the primary assisted reproductive technique for fertilizing oocytes outside the female reproductive system. Sleep disorders are likely to affect infertility and its treatment. The present study was to investigate the relationship between sleep disturbances and IVF outcomes in infertile women.

**Methods:**

Databases, including PubMed, Embase, Cochrane Library, ProQuest, and Web of Science, were searched for the relevant articles published up to September 2023. The Newcastle–Ottawa Scale was used to assess the methodological quality of the included studies. Moreover, the random and fixed effects models using the STATA (v.11) software program presented the odds ratio with a 95% confidence interval (CI). Ultimately, a funnel plot was recruited to examine the sensitivity analysis.

**Results:**

During the initial search, 426 articles were retrieved, and ultimately, nine studies remained for systematic review, and among them, four studies entered into the meta‐analysis (two cohort and two cross‐sectional studies). According to the fixed effects model of the cross‐sectional studies, the association between sleep quality and pregnancy rate was not confirmed (OR = 0.94; 95% CI = 0.81–1.07). Regarding the fixed effects model of included cohort studies, the results indicated an association between sleep quality and the pregnancy rate (OR = 1.08; 95% CI = 1.03–1.14). According to the random effects model of the cross‐sectional studies, there was no significant association between sleep quality and pregnancy rate (OR = 0.82; 95% CI = 0.37–1.26). Regarding the random effect analysis of included cohort studies, the results indicated an association between sleep quality and the pregnancy rate (OR = 1.08; 95% CI = 1.03–1.14). Based on the total fixed effect analysis of the cross‐sectional and cohort studies, the results also showed a significant association between sleep quality and pregnancy rate among infertile women (OR = 1.06; 95% CI = 1.01–1.11). In contrast, according to the random effect of the total studies, this association was not confirmed (OR = 1.02; 95% CI = 0.90–1.14).

**Conclusion:**

Although the meta‐analysis of the cohort studies showed a significant association between sleep quality and IVF outcomes, such as pregnancy rate, due to the novelty of the subject, more studies have not been published, and this study is considered a preliminary meta‐analysis. Therefore, more studies with a strong methodology need to assess the relationship between sleep disorders and IVF outcomes.

## Graphical abstract

According to the random effect model of the cross‐sectional studies, there was no significant association between sleep quality and pregnancy rate (OR = 0.82, 95% CI = [0.37–1.26]). Regarding the random effect analysis of included cohort studies, the results indicated an association between sleep quality and the pregnancy rate (OR = 1.08, 95% CI = [1.03, 1.14]).

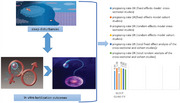

**Abbreviations**
AMH: anti‐Müllerian hormone
ART: assisted reproductive technology
CI: confidence interval
ET: embryo transfer
FEM: fixed effects model
FSH: follicle‐stimulating hormone
*I* ^2^
: *I* squared
IRR: incidence rate ratio
IVF: in vitro fertilization
MeSH: Medical Subject Headings
NOS: Newcastle–Ottawa Scale
OR: odds ratio
PECO
: population, exposure, comparison, and outcomes
PRISMA: Preferred Reporting Items for Systematic Reviews and Meta‐Analyses
PSQI: Pittsburgh Sleep Quality Index
REM: random effects model
SDB: sleep‐disordered breathing

## 1 Introduction

Infertility means failure to achieve a clinical pregnancy after 12 months of regular unprotected intercourse or reproductive disorders (Kesharwani et al. 2022). The prevalence rate of infertility is estimated at 20% worldwide and 25% among couples in developing countries (Dabbagh Rezaeiyeh et al. 2022). According to global estimates, over half of those with fertility problems seek medical care, less than a quarter receive treatments, and approximately 3% undergo assisted reproductive technology (ART) techniques (Coussa, Hasan, and Barber 2020). In vitro fertilization (IVF), with a success rate of 35%, accordingly accounts for 99% of the ART‐related procedures (Guven, Cayir, and Borekci 2020) as the primary method practiced for this purpose, in which the oocytes are fertilized outside the female reproductive system, whose application is steadily escalating due to its safe prenatal outcomes (De Geyter 2019; Levine et al. 2017; Lv et al. 2021). Studies have thus far shown that the different stages of infertility treatment create psychological distress (Ilyin et al. 2019; Sharma and Shrivastava 2022; Wilkins, Warnock, and Serrano 2010; Ying, Wu, and Loke 2016). In other words, women living with infertility may experience adverse emotional reactions, such as low self‐esteem, body image disturbance, decreased marital satisfaction, depression, and anxiety (Kostić and Mitrović 2022; Ražić Pavičić et al. 2022). In addition, studies showed that infertility and its treatments negatively affect couples’ sleep quality (Du et al. 2022; Hvidt et al. 2020).

Sleep quality is defined as an individual's self‐satisfaction with all aspects of the sleep experience, such as sleep efficiency, latency, duration, and wake after sleep onset (Nelson, Davis, and Corbett 2022). Sleep disturbances can also include sleep interruption, irregular sleep–wake syndrome, excessively long or short sleep time, circadian rhythm disorders, hypoxia, or sleep apnea (Koochaksaraei et al. 2022; Moursund 2015).

Although women undergoing IVF may be thus vulnerable to sleep disturbances associated with stress and anxiety throughout this procedure (Goldstein et al. 2017; Pimolsri et al. 2021), the data on the effects of sleep on nonpsychological outcomes of IVF are still limited (J. Lin, Lin, and Chueh 2014). In this respect, according to a Chinese study conducted in 2022, there was a significant relationship between sleep quality and fertility outcomes in women undertaking IVF (Liu et al. 2022). Also, Caetano et al. (2021), in a review of 33 articles, reflected on the relationship between sleep and reproductive health in couples. Still, no definitive results were obtained despite the volume of the studies. A recent survey of nearly 7000 women also found that self‐reported difficulty sleeping was associated with increased time to conception, and there was a weak correlation between short sleep duration and reduced fertility (Fernandez et al. 2020). Y. Lin, Chueh, and Lin (2016) additionally reported that 23% and 46% of women undergoing IVF had experienced sleep disorders during oocyte retrieval and implantation (Y. Lin, Chueh, and Lin 2016). Besides, Goldstein et al. (2017) showed that 57%, 43%, and 29% of women receiving IVF had sleep disturbances before treatment, stimulation, and embryo transfer (ET), respectively. They also found that hormones, such as the anti‐Müllerian hormone (AMH), follicle‐stimulating hormone (FSH), and total sleep time (TST), could influence patient sleep at some stage in the oocyte retrieval process before treatment (Goldstein et al. 2017). In this line, the results of a study investigated the relationship between sleep and pregnancy outcomes in women undergoing IVF, but no statistically significant association was observed (Shu et al. 2022).

Therefore, there is a need to better perceive the sleep conditions of women receiving IVF with increased emotional distress to improve ART outcomes. In recent years, there has been a growing interest in research on sleep quality among infertile women (Eisenberg et al. 2021; Ibrahim et al. 2023; Lim et al. 2021). Examining sleep quality during infertility treatments is thus of utmost importance because of its significant relationship with anxiety and stress. In other words, sleep disturbances can be a sign of mental health disorders, which affect clinical outcomes in practice (Calvo et al. 2023; Rooney and Domar 2022). In addition, evidence shows that sleep plays a leading role in many medical conditions (Lane et al. 2023; Lechat et al. 2022; Ullah and Tamanna 2023). However, the relationship between sleep and infertility treatment outcomes is still unclear, and studies are limited or do not report conclusive results. Given the position of IVF as an infertility treatment and the mutual impacts of mental health (specifically anxiety, depression, and sleep disorders) and IVF, no systematic review investigating the relationship between sleep disturbances and IVF outcomes was found, to the best of the author's knowledge. Hence, this study aimed to examine the relationship between sleep disorders (namely, decreased or increased sleep duration, insomnia, sleep quality, and sleep apnea) and IVF outcomes in infertile women.

## 2 METHODS

### 2.1 Focused Research Question

This systematic review and meta‐analysis study was designed and written based on the Preferred Reporting Items for Systematic Reviews and Meta‐Analyses (PRISMA) (Page et al. 2021). The present study was compiled according to the AMSTAR checklist and was registered on PROSPERO (CRD42023453948).

In addition, the population, exposure, comparison, and outcomes (PECO) framework was utilized to formulate the following question: “Do sleep disturbances affect IVF outcomes?”

The preprint of this manuscript is published and is available on https://assets‐eu.researchsquare.com/files/rs‐2948771/v1/5cff94db‐a63b‐4e3e‐a227‐b27b69d9c89a.pdf?c=1689665393.

### 2.2 Eligibility Criteria

The inclusion criteria for this systematic review and meta‐analysis were the original observational studies (i.e., the cohort, cross‐sectional, and case‐control ones) exploring the association between sleep disturbances and IVF outcomes, using specific measures of sleep time, including the Pittsburgh Sleep Quality Index (PSQI), interviews, or other questionnaires, with no language, time, and geographic limits. On the other hand, review articles, abstracts, and qualitative and interventional studies were excluded.

### 2.3 Protocol for Article Search and Data Extraction

Two authors (i.e., F.H. and M.A.) searched the articles without language limits in the databases of PubMed, Embase, the Cochrane Library, ProQuest, and Web of Science published up to September 2023. The search strategy in databases is presented in Table 1. Accordingly, the studies relevant to the research question were retrieved using the Medical Subject Headings (MeSH) descriptors of “obstructive sleep apnea, sleep quality, sleep disorders, sleep behavior, sleep duration, sleep habit, sleep pattern, insomnia, infertility treatment, infertility management, assisted human reproduction, in vitro fertilization, and IVF.”

**TABLE 1:** Search strategy.

Databases	Search strategy
PubMed	(“Sleep pattern” [tiab] or “obstructive sleep apnea” [tiab] or “sleep quality” [tiab] or “sleep disorder” [tiab] or “sleep behavior” [tiab] or “sleep duration” [tiab] or “sleep habit” [tiab] or “insomnia” [tiab]) and (“infertility treatment” [tiab] or “infertility management” [tiab] or “assisted reproduction” [tiab] or “in vitro fertilization” [tiab])
Embase	“obstructive sleep apnea” AND “infertility treatment”—“obstructive sleep apnea” AND “infertility management”—“obstructive sleep apnea” AND “assisted reproduction”—“obstructive sleep apnea” AND “in vitro fertilization” “sleep quality” AND “infertility treatment”—“sleep quality” AND “infertility management”—“sleep quality” AND “assisted reproduction”—“sleep quality” AND “in vitro fertilization” “sleep disorder” AND “infertility treatment”—“sleep disorder” AND “infertility management”—“sleep disorder” AND “assisted reproduction”—“sleep disorder” AND “in vitro fertilization” “sleep behavior” AND “infertility treatment”—“sleep behavior” AND “infertility management”—“sleep behavior” AND “assisted reproduction”—“sleep behavior” AND “in vitro fertilization” “sleep duration” AND “infertility treatment”—“sleep duration” AND “infertility management”—“sleep duration” AND “assisted reproduction”—“sleep duration” AND “in vitro fertilization” “sleep habit” AND “infertility treatment”—“sleep habit” AND “infertility management”—“sleep habit” AND “assisted reproduction”—“sleep habit” AND “in vitro fertilization” “sleep insomnia” AND “infertility treatment”—“sleep insomnia” AND “infertility management ”—“sleep insomnia” AND “assisted reproduction”—“sleep insomnia” AND “in vitro fertilization”
Cochrane Library	“obstructive sleep apnea” AND “infertility treatment”—“obstructive sleep apnea” AND “infertility management”—“obstructive sleep apnea” AND “assisted reproduction”—“obstructive sleep apnea” AND “in vitro fertilization” “sleep quality” AND “infertility treatment”—“sleep quality” AND “infertility management”—“sleep quality” AND “assisted reproduction”—“sleep quality” AND “in vitro fertilization” “sleep disorder” AND “infertility treatment”—“sleep disorder” AND “infertility management”—“sleep disorder” AND “assisted reproduction”—“sleep disorder” AND “in vitro fertilization” “sleep behavior” AND “infertility treatment”—“sleep behavior” AND “infertility management”—“sleep behavior” AND “assisted reproduction”—“sleep behavior” AND “in vitro fertilization” “sleep duration” AND “infertility treatment”—“sleep duration” AND “infertility management”—“sleep duration” AND “assisted reproduction ”—“sleep duration” AND “in vitro fertilization” “sleep habit” AND “infertility treatment”—“sleep habit” AND “infertility management”—“sleep habit” AND “assisted reproduction”—“sleep habit” AND “in vitro fertilization” “sleep insomnia” AND “infertility treatment”—“sleep insomnia” AND “infertility management ”—“sleep insomnia” AND “assisted reproduction”—“sleep insomnia” AND “in vitro fertilization”
ProQuest	“obstructive sleep apnea” AND “infertility treatment”—“obstructive sleep apnea” AND “infertility management”—“obstructive sleep apnea” AND “assisted reproduction”—“obstructive sleep apnea” AND “in vitro fertilization” “sleep quality” AND “infertility treatment”—“sleep quality” AND “infertility management”—“sleep quality” AND “assisted reproduction”—“sleep quality” AND “in vitro fertilization” “sleep disorder” AND “infertility treatment”—“sleep disorder” AND “infertility management”—“sleep disorder” AND “assisted reproduction”—“sleep disorder” AND “in vitro fertilization” “sleep behavior” AND “infertility treatment”—“sleep behavior” AND “infertility management”—“sleep behavior” AND “assisted reproduction ”—“sleep behavior” AND “in vitro fertilization” “sleep duration” AND “infertility treatment”—“sleep duration” AND “infertility management”—“sleep duration” AND “assisted reproduction ”—“sleep duration” AND “in vitro fertilization” “sleep habit” AND “infertility treatment”—“sleep habit” AND “infertility management”—“sleep habit” AND “assisted reproduction”—“sleep habit” AND “in vitro fertilization” “sleep insomnia” AND “infertility treatment”—“sleep insomnia” AND “infertility management”—“sleep insomnia” AND “assisted reproduction”—“sleep insomnia” AND “in vitro fertilization”
Web of Science	“obstructive sleep apnea” AND “infertility treatment”—“obstructive sleep apnea” AND “infertility management”—“obstructive sleep apnea” AND “assisted reproduction”—“obstructive sleep apnea” AND “in vitro fertilization” “sleep quality” AND “infertility treatment”—“sleep quality” AND “infertility management”—“sleep quality” AND “assisted reproduction”—“sleep quality” AND “in vitro fertilization” “sleep disorder” AND “infertility treatment”—“sleep disorder” AND “infertility management”—“sleep disorder” AND “assisted reproduction”—“sleep disorder” AND “in vitro fertilization” “sleep behavior” AND “infertility treatment”—“sleep behavior” AND “infertility management”—“sleep behavior” AND “assisted reproduction”—“sleep behavior” AND “in vitro fertilization” “sleep duration” AND “infertility treatment”—“sleep duration” AND “infertility management”—“sleep duration” AND “assisted reproduction”—“sleep duration” AND “in vitro fertilization” “sleep habit” AND “infertility treatment”—“sleep habit” AND “infertility management”—“sleep habit” AND “assisted reproduction”—“sleep habit” AND “in vitro fertilization” “sleep insomnia” AND “infertility treatment”—“sleep insomnia” AND “infertility management ”—“sleep insomnia” AND “assisted reproduction”—“sleep insomnia” AND “in vitro fertilization”

To minimize the possibility of bias, two authors (i.e., F.H. and M.A.) independently screened the titles and abstracts of the articles using the abovementioned keywords. In case of disagreement, they discussed the article with a third author (viz., Z.S.). Then, they independently evaluated the full texts of the studies selected based on their titles and abstracts. After the initial electronic search, the references of the selected articles were manually searched to further identify the potentially relevant ones.

### 2.4 Methodological Quality

The Newcastle–Ottawa Scale (NOS) was employed to improve the methodological quality of the studies included in this review. This scale allocates a total score of 9, the highest quality, to cohort studies: 4 scores for participant selection, 2 for comparability, and 3 for outcome assessment. NOS allocates a total score of 10, the highest quality, to cross‐sectional studies: 5 scores for selecting participants, 2 scores for comparability, and 3 for outcome assessment. In which higher scores indicated better quality. Given that, two authors (i.e., F.H. and M.A.) independently assessed the quality of all studies (Table 2) (Wells et al. 2022).

**TABLE 2:** Assessment of the methodological quality of eligible studies via the NOS.

No.	Studies	Selection	Comparability	Outcome	Total score
1	Philipsen et al. (2022)	2	2	2.5	6.5
2	Pimolsri et al. (2021)	2.5	2	3	7.5
3	Reschini et al. (2022)	2	2	2.5	6.5
4	Goldstein et al. (2017)	3	2	2.5	7.5
5	Liu et al. (2022)	3	2	2	7
6	Yao et al. (2022)	3	2	2	7
7	Walter et al. (2022)	3	2	2.5	7.5
8	Mengye et al. (2022)	3	2	2.5	7.5
9	Q. Li et al. (2023)	3	2	2.5	7.5

### 2.5 Data Extraction and Management

Two researchers (i.e., F.H. and M.A.) independently extracted and entered the data into a predetermined form. They further discussed their disagreement with a third researcher and contacted the corresponding authors of studies in case of ambiguous information. The extracted data included authors’ names, year of publication, setting, objectives, sample size and participants, study design, instruments, primary data, and main results (Table 3).

**TABLE 3:** The characteristics of the included studies.

No.	Article specifications (author's name, year of publication, setting)	Study design	Sample size and participants	Instruments	Main results
1	Philipsen et al. (2022), Denmark (	Cross‐sectional	163 women and 132 partners	PSQI	Women with good sleep quality had higher clinical pregnancy rates (PSQI ≤ 5 = 72.7%, PSQI 6–10 = 52.6%, and PSQI ≥ 11 = 42.3%).
2	Pimolsri et al. (2021), USA	Cohort	48 women	Wrist‐worn actigraphy	Women with longer sleep duration had a lower possibility of an uncompleted IVF cycle (OR = 0.88; 95% CI = 0.78–1.00). Women with later sleep midpoint and later bedtime had a higher likelihood of experiencing an uncompleted cycle compared to those with earlier midpoint and earlier bedtime (OR = 1.24; 95% CI = 1.09–1.40) and (OR = 1.33; 95% CI = 1.17–1.53), respectively.
3	Reschini et al. (2022), Italy	Cross‐sectional	263 women	PSQI	There was a significant difference regarding the PSQI score in pregnant and nonpregnant women (*p* = 0.004). The crude and adjusted OR of pregnancy in women with a PSQI > 5 (impaired sleep quality) was 0.46 (95% CI = 0.25–0.86; *p* = 0.02) and 0.50 (95% CI = 0.26–0.94; *p* = 0.03), respectively.
4	Goldstein et al. (2017), USA	Cohort	22 women	PSQI	A linear significant association was found between total sleep time and oocytes retrieved (adjusted *R* ^2^ = 0.40; *p* = 0.03).
5	Liu et al. (2022), China	Cohort	7847 women	PSQI	Women with good sleep quality had higher clinical pregnancies than those with poor sleep quality (69.3% vs. 65.1%) and live birth rates (50.5% vs. 45.7%).
6	Yao et al. (2022), China	Cohort	1276 women	PSQI	The study showed a positive association between the number of quality embryos, fertilization rate, and poor subjective sleep quality (*p* = 0.02 and 0.03, respectively).
7	Walter et al. (2022), USA	Cohort	30 women	Home‐based wireless, wearable sensors	Sleep‐disordered breathing with each severity showed a reduction of 77% in pregnancy rate and live birth.
8	Mengye et al. (2022), China	Cohort	1344 women	PSQI	The poor sleep quality in mothers during the second trimester was significantly associated with SGA among female infants (OR = 3.03; 95% CI = 1.04–9.71; *p* = 0.044), while there was no significant association between the poor sleep quality and the male infant birth weight (OR = 1.64; 95% CI = 0.68–3.99; *p* = 0.271).
9	Q. Li et al. (2023), China	Cohort	1002 women	PSQI	There was a significant negative association between the sleep quality and the quality of retrieved oocytes incidence rate ratio (IRR = 0.85; 95% CI = 0.72–0.99; *p* = 0.038) and fertilization rates (IRR = 0.96; 95% CI = 0.92–1.00; *p* = 0.034).

Abbreviations: CI, confidence interval; OR, odds ratio; PSQI, Pittsburgh Sleep Quality Index; SGA, small for gestational age.

### 2.6 Data Synthesis

The data heterogeneity was evaluated using the *I*‐square (*I* ^2^) index. Suppose, the results were 0%–25%, the *I* ^2^ index was then categorized into 25%–75% and > 75%, which were considered low, moderate, and high heterogeneity. If the heterogeneity test was not significant in the meta‐analysis (*p* > 0.05), the results of the fixed effects model (FEM) were used to output the odds ratios (ORs) and their 95% confidence interval (CI); if not, they reported the results of the random effects model (REM) utilizing the STATA (v.11) software. Moreover, sensitivity analysis was practiced to determine the impact of each study on its final results. It showed how much each study could contribute to the overall outcomes of the ORs and the robustness of the findings for the decisions made in this process.

We approximated the standard errors via the *p* values and 95% CI. Based on the *p* value, we first converted the two‐sided *p* values into one‐sided *p* values by dividing them by 2. Then, the *p* values were converted to the corresponding *z* values. The obtained *z* values are the test statistics calculated by *z* = ln(OR)/SE. So, it follows that SE = ln(OR)/z. We also calculated SE from 95% CI using the below formula:

$$\mathrm{Lower}\;\mathrm{limit}=\ln\left(\mathrm{lower}\;\mathrm{confidence}\;\mathrm{limit}\;\mathrm{given}\;\mathrm{for}\;\mathrm{RR}\right),$$

$$\mathrm{Upper}\;\mathrm{limit}=\ln\left(\mathrm{upper}\;\mathrm{confidence}\;\mathrm{limit}\;\mathrm{given}\;\mathrm{for}\;\mathrm{RR}\right),$$

SE=(upperlimit−lowerlimit)/3.92.

In addition, Liu et al. (2022) reported relative risk (RR) instead of OR. We converted RR to OR using the formula below, on which p is the risk of the control group.

$$OR=\frac{\mathrm{RR}\;\left(1\;-\;p\right)}{1\;-\;\left(p\;\times \;\mathrm{RR}\right)}$$

## 3 RESULTS

### 3.1 Search Results

**FIGURE 1:**
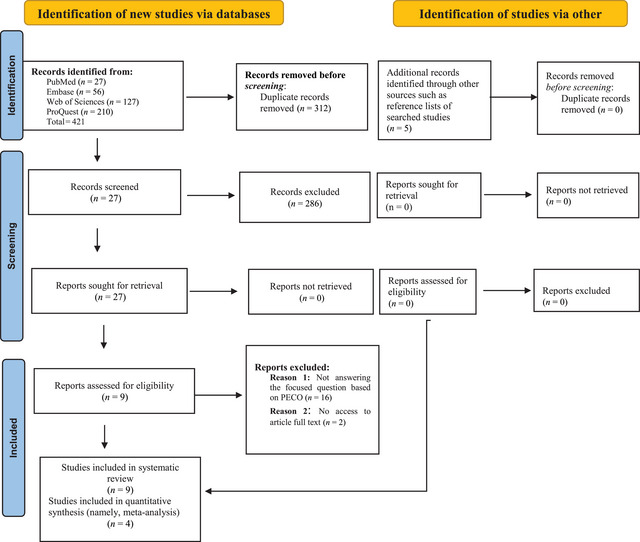
The PRISMA diagram for the search of records and study selection.

In total, 426 studies were retrieved during the initial search. During the screening stage, 27 articles were selected for full‐text reviews after removing the duplicates and examining their titles and abstracts. Then, 16 articles that did not address the focused research question based on the PECO and two cases whose full texts were unavailable were removed. Finally, nine studies remained for systematic review (seven cohort and two cross‐sectional studies). Four studies entered into the meta‐analysis (two cohorts and two cross‐sectional studies). Figure 1 demonstrates the study identification diagram according to the PRISMA guidelines with the reasons for excluding those after reading their full texts.

### 3.2 Sleep Quality

In the review, five studies evaluated the relationship between sleep quality and different dimensions of IVF. Philipsen et al. (2022) reported that poor sleep quality was typical in couples undergoing the IVF techniques (91% of participants had PSQI scores > 5, indicating poor sleep quality). Poor sleep quality could also affect women and their partners. Moreover, they revealed a significant negative relationship between sleep quality and symptoms of depression and anxiety (*p* < 0.001), suggesting a possible correlation between sleep quality and pregnancy outcomes (PSQI ≤ 5 = 72.7%, PSQI 6–10 = 52.6%, and PSQI ≥ 11 = 42.3%) (Philipsen et al. 2022). Similarly, Reschini et al. (2022) demonstrated that poor sleep quality was prevalent in women experiencing IVF, affecting the success of this procedure. Statistically significant differences emerged for the PSQI score, the median (interquartile range) in pregnant and nonpregnant women being 4 (3–5) and 5 (3–7), respectively (*p* = 0.004). The crude and adjusted OR of pregnancy in women with a PSQI > 5 (indicating impaired sleep quality) was 0.46 (95% CI = 0.25–0.86; *p* = 0.02) and 0.50 (95% CI = 0.26–0.94; *p* = 0.03), respectively (Reschini et al. 2022). In addition, Liu et al. (2022) concluded that sleep quality and timing before ET were associated with the outcomes, and good sleep quality was an influential factor for pregnancy, such as conception and live birth (people with good sleep quality showed a higher clinical pregnancy (69.3% vs. 65.1%) and more live birth rates (50.5% vs. 45.7%) (Liu et al. 2022). Likewise, Mengye et al. (2022) established that poor sleep quality in the first and second trimesters could negatively influence the weight of the IVF female infants (OR = 3.03; 95% CI = 1.04–9.71; *p* = 0.044) and underlined that early screening and interventions focused on sleep quality improvement could reduce the adverse consequences of poor sleep. They also reiterated that sleep interventions and psychological counseling during pregnancy might be helpful for fetal health (Mengye et al. 2022). In the study by Q. Li et al. (2023), which investigated the sleep characteristics before ART and reproductive outcomes among Chinese infertile women, the results indicated that sleep disorders such as poor sleep quality and efficiency decreased quality and quantity of sleep negatively affect the quality of retrieved oocytes incidence rate ratio (IRR = 0.85; 95% CI = 0.72–0.99; *p* = 0.038) and fertilization rates (IRR = 0.96; 95% CI = 0.92–1.00; *p* = 0.034). In addition, the poor sleep quality before the ART process had a negative treatment effect on infertility treatment (Q. Li et al. 2023).

### 3.3 Sleep Duration

Four studies assessed the sleep duration among infertile women; Pimolsri et al. (2021) demonstrated a significant relationship between short sleep duration and midpoint and later sleep time with the lower odds of an uncompleted IVF cycle. Longer sleep duration was associated with lower odds of an uncompleted IVF cycle (OR = 0.88; 95% CI = 0.78–1.00; per 20‐min increment of increased sleep duration). Women with later sleep midpoint and later bedtime had higher odds of uncompleted cycle relative to those with earlier midpoint and earlier bedtime, OR = 1.24; 95% CI = 1.09–1.40 and OR = 1.33; 95% CI = 1.17–1.53, respectively, for 20‐min increments. In addition, Goldstein et al. (2017) emphasized the role of sleep duration in ovarian response and IVF outcomes (*R* ^2^ = 0.40; *p* = 0.03). In this line, Yao et al. (2022) showed that short and disturbed sleep were associated with decreased oocyte quantity and quality of 11.5% (95% CI = −21.3% to −0.48%) and 11.9% (95% CI = −22.4% to −0.03%), respectively, and long sleep duration was related to the reduced chance of clinical pregnancy (OR = 0.65; 95% CI = 0.44–0.98). In the study by Q. Li et al. (2023), the results demonstrated that there was a significant association between sleep duration and retrieved oocyte rates (OR = 1.04; 95% CI = 1.00–1.09) among Chinese infertile women.

### 3.4 Sleep‐Disordered Breathing

In the present study, one study evaluated the relationship between sleep‐disordered breathing (SDB) and different outcomes of IVF. Walter et al. (2022) indicated that the overall rate of SDB of any severity was 57%. Of the 29 patients undergoing an ET, clinical pregnancy and live birth occurred in 35% of women with SDB compared to 58% without SDB (*p* = 0.22). After adjusting for age, SDB reduced pregnancy rates but was not statistically significant (AOR 0.23; 95% CI = 0.04–1.5; *p* = 0.12). Though polycystic ovary syndrome was associated with higher rates of SDB, it was not independently associated with lower pregnancy rates. In addition, they presented SDB as a new modifiable risk factor for the tremendous success of this procedure (Walter et al. 2022).

### 3.5 Meta‐Analysis of the Included Studies

**FIGURE 2:**
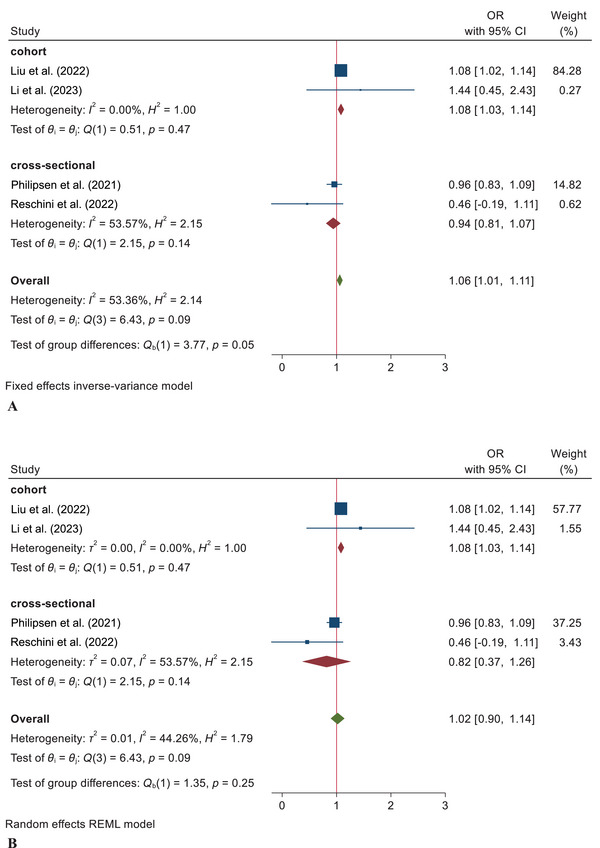
Forest plot for a relationship between sleep quality and clinical pregnancy rate combining reported ORs in studies with 95% CI based on the *I* ^2^ index: fixed effects model (A) and random effects model (B).

Four articles were entered into this meta‐analysis, including two cross‐sectional and two cohort studies. In Figure 2A, according to the FEM of the cross‐sectional studies, the association between sleep quality and pregnancy rate was not confirmed (OR = 0.94; 95% CI = 0.81–1.07). Regarding the FEM of included cohort studies, the results indicated an association between sleep quality and the pregnancy rate (OR = 1.08; 95% CI = 1.03–1.14).

In Figure 2B, according to the REM of the cross‐sectional studies, there was no significant association between sleep quality and pregnancy rate (OR = 0.82; 95% CI = 0.37–1.26). Regarding the random effect analysis of included cohort studies, the results indicated an association between sleep quality and the pregnancy rate (OR = 1.08; 95% CI = 1.03–1.14). Based on the total fixed effect analysis of the cross‐sectional and cohort studies, the results also showed a significant association between sleep quality and pregnancy rate among infertile women (OR = 1.06; 95% CI = 1.01–1.11). In contrast, according to the random effect of the total studies, this association was not confirmed (OR = 1.02; 95% CI = 0.90–1.14).

## 4 Discussion

This systematic review and meta‐analysis was the first attempt to examine the relationship between sleep disturbances and IVF outcomes. The study findings illustrated that sleep disorders were more common in women undergoing the IVF techniques than in the general population, affecting their outcomes, such as pregnancy, oocyte retrieval, ovulation cycle, live birth, and fetal weight. The results of the meta‐analysis showed that according to the cohort‐included studies, there was a significant association between sleep quality and pregnancy rate. In contrast, this association was not confirmed according to the cross‐sectional studies. Consistently, Caetano et al. (2021) conducted a systematic review to evaluate the effect of sleep parameters on male and female reproductive functions. They accordingly considered 33 articles, of which six cases investigated the relationship between sleep and IVF. The review of these studies showed that sleep disturbances during oocyte retrieval were higher in women receiving IVF compared with the general population (Caetano et al. 2021). This result is consistent with the results of our study.

The included studies in the systematic review section of this present study showed a significant relationship between sleep duration and IVF outcomes (uncompleted IVF cycle, ovarian response, decreased oocyte quantity and quality, clinical pregnancy). In a review conducted by Zhao et al. (2022), an inverse relationship was observed between limited sleep duration (≤ 7 h) and fertility (OR = 0.92; 95% CI = 0.84–1.00; *I* ^2^ = 0%) compared to 8 h of sleep. The findings are consistent with the results of current study. The pregnancy rate was significantly higher in the moderate sleep group than in the extended sleep group (52.6% [234/445] vs. 42.9% [39/91]; P = 0.045). The pregnancy rate of the group with moderate sleep was also higher than that of the group who slept short (52.6% [234/445] vs. 45.8% [55/120]; P = 0.090). Therefore, the study's results showed that adequate sleep has a beneficial effect on the secretion of reproductive hormones and may be helpful for fertility and IVF results (Park et al. 2013).

Also, the results of the cohort studies in the meta‐analysis section of this present study showed a significant association between sleep quality and IVF outcomes, such as pregnancy outcomes, conception, live birth, and higher clinical pregnancy. In a study conducted by P. Li and Wu (2015), poor sleep quality is considered an independent risk factor for premature ovarian failure (OR = 3.72; 95% CI = 1.17–11.81). In addition, in a study conducted by Özçelik et al. (2023), the quality of sleep in infertile people was lower than in the general population (*p* <  0.001). Also, in another study conducted by Llaneza, Llaneza, and Fernandez‐Ferrera (2017), serum estradiol levels at the end of ovarian stimulation and the number of oocytes retrieved were lower in women with poor sleep quality (*p* = 0.018 and *p* = 0.013), and poor sleep quality was associated with poor ovarian response (*R* ^2^ = 0.846; *p* = 0.048). The results of this study are also inconsistent with our investigation. In addition, large‐scale research has been suggested to determine how sleep and fertility are linked and how sleep could be a helpful modifiable target in infertility management. In a narrative review, Spaggiari et al. (2022) correspondingly determined the relationship between human sleep habits and fertility, exploring the relationship between women's sleep disorders (e.g., sleep quality, sleep duration, and sleep habits) and their reproductive functions, male reproductive functions, natural conception, and assisted reproduction. The study results then established that reduced sleep duration could negatively influence the parameters related to human fertility. They have further shown that numerous mechanisms, such as adrenal activation, circadian dysregulation, and genetic effects, were associated with sleep disturbances and reproductive health (Spaggiari et al. 2022). Besides, Szkodziak, Krzyżanowski, and Szkodziak (2020) assessed the impact of mental disorders on male and female fertility as the causes of idiopathic infertility. Sleep disturbances have been thus introduced as mental disorders affecting male and female reproductive health (Szkodziak, Krzyżanowski, and Szkodziak 2020). According to a recent study, sleep disturbances and low reproductive health could lead to infertility and then shape treatment outcomes.

### 4.1 Strength and Limitations

This study is the first systematic review and meta‐analysis that assessed the association between sleep disturbance and IVF outcomes. However, this study had some limitations. First, based on the inclusion and exclusion criteria, the studies included in the study were limited and had a relatively low sample size, indicating the lack of sufficient quality studies in this field. Moreover, to the best of the authors’ knowledge, the review of the existing sources revealed that no study had already investigated the maternal and fetal consequences of IVF, such as early or late abortion, stillbirth, preterm birth, bleeding, postpartum depression, and others.

One of the strengths of this systematic review and meta‐analysis was addressing women undergoing IVF, considering the relatively high prevalence of infertility in society. However, theoretically, a meta‐analysis with two studies is possible; it is better to have more studies in this regard (Myung 2023). Due to the novelty of the subject, more studies have not been published, and this study is considered a preliminary meta‐analysis. Therefore, the present study is hoped to be a turning point that encourages more extensive research.

### 4.2 Implications in Practice

Physicians and reproductive health service providers accordingly need to be sensitive and well‐informed about the study and diagnosis of sleep disturbances in infertile women as a vulnerable group. In conclusion, it would be possible to promote the quality of life and mental health of those undergoing infertility treatments by improving sleep disorders, thereby raising the potential of natural pregnancy and boosting the outcomes of infertility treatments.

## 5 Conclusion

The results of the meta‐analysis showed that according to the cohort‐included studies, there was a significant association between sleep quality and pregnancy rate. In contrast, this association was not confirmed according to the cross‐sectional studies. As a low volume of studies was included in the meta‐analysis, high‐quality observational studies are needed to confirm the relationship between sleep disorder and IVF outcomes with high‐certainty evidence. Since infertility and the use of ART techniques, especially IVF, are expanding worldwide, it is necessary to conduct more comprehensive studies in infertility clinics to investigate the effect of sleep disturbances on various IVF outcomes.

Regarding the value of reproductive health and the treatment of infertile people, more studies followed by systematic reviews and stronger meta‐analyses with higher volumes of articles are required to confirm the relationship between different types of sleep disturbances and IVF outcomes in infertile women. Suppose cross‐sectional and cohort studies prove the relationship between sleep disorders and IVF outcomes. In that case, it is necessary to conduct interventional studies to find whether improved sleep positively affects IVF outcomes.

## Author Contributions

**Farangis Habibi**: writing–original draft, writing–review and editing, data curation, methodology. **Roya Nikbakht**: formal analysis, methodology. **Shayesteh Jahanfar**: writing–review and editing, supervision, conceptualization. **Mohammad Ahmadi**: Investigation. **Maryam Eslami**: visualization, investigation. **Marzieh Azizi**: writing–review and editing, data curation. **Zohreh Shahhosseini**: supervision, conceptualization.

## Conflicts of Interest

The authors declare no conflicts of interest.

### Peer Review

The peer review history for this article is available at https://publons.com/publon/10.1002/brb3.70293.

## Data Availability

All data have been presented in the manuscript.
